# Developmental Exposure to Second-Hand Smoke Increases Adult Atherogenesis and Alters Mitochondrial DNA Copy Number and Deletions in apoE^−/−^ Mice

**DOI:** 10.1371/journal.pone.0066835

**Published:** 2013-06-25

**Authors:** Jessica L. Fetterman, Melissa Pompilius, David G. Westbrook, Dale Uyeminami, Jamelle Brown, Kent E. Pinkerton, Scott W. Ballinger

**Affiliations:** 1 The University of Alabama at Birmingham, Division of Molecular and Cellular Pathology, Birmingham, Alabama, United States of America; 2 University of California at Davis, Center for Health and Environment, Davis, California, United States of America; 3 Department of Pathology, Division of Molecular and Cellular Pathology, 535 BMR2, 1720 2nd Ave S, Birmingham; University of Medicine and Dentistry of New Jersey, United States of America

## Abstract

Cardiovascular disease is a major cause of morbidity and mortality in the United States. While many studies have focused upon the effects of adult second-hand smoke exposure on cardiovascular disease development, disease development occurs over decades and is likely influenced by childhood exposure. The impacts of *in utero* versus neonatal second-hand smoke exposure on adult atherosclerotic disease development are not known. The objective of the current study was to determine the effects of *in utero* versus neonatal exposure to a low dose (1 mg/m^3^ total suspended particulate) of second-hand smoke on adult atherosclerotic lesion development using the apolipoprotein E null mouse model. Consequently, apolipoprotein E null mice were exposed to either filtered air or second-hand smoke: (i) *in utero* from gestation days 1–19, or (ii) from birth until 3 weeks of age (neonatal). Subsequently, all animals were exposed to filtered air and sacrificed at 12–14 weeks of age. Oil red-O staining of whole aortas, measures of mitochondrial damage, and oxidative stress were performed. Results show that both *in utero* and neonatal second-hand smoke exposure significantly increased adult atherogenesis in mice compared to filtered air controls. These changes were associated with changes in aconitase and mitochondrial superoxide dismutase activities consistent with increased oxidative stress in the aorta, changes in mitochondrial DNA copy number and deletion levels. These studies show that *in utero* or neonatal exposure to second-hand smoke significantly influences adult atherosclerotic lesion development and results in significant alterations to the mitochondrion and its genome that may contribute to atherogenesis.

## Introduction

Second-hand tobacco smoke (SHS) is a major cardiovascular disease (CVD) risk factor that significantly increases individual CVD risk [1]–[4]. While CVD is often thought of as an adult disease, the Pathobiological Determinants of Atherosclerosis in Youth (PDAY) and Fate of Early Lesions in Children (FELIC) studies clearly show that atherogenesis can begin early in life based upon the presence of vascular fatty streaks in children and young adults, and that cigarette smoke exposure associated with increased lesion formation in these individuals [5]–[9]. It has also been estimated that 59% of children between the ages of 3 and 11 years of age have been exposed to SHS based upon serum cotinine levels [10]. Finally, 13.2% of women continue to smoke during pregnancy [11], [12]. In this regard, it has been shown that prenatal (*in utero*) exposure to 10 mg/m^3^ TSP (total suspended particulate) SHS increased adult atherosclerotic lesion development in a gender biased fashion in mice (males more susceptible) [13], [14], and more recently, that perinatal exposure to tenfold lower SHS dose (1 mg/m^3^ TSP) in non-human primates results in significant changes in endothelial pathology reminiscent of atherosclerosis [15]. Another feature of these studies was that mitochondrial damage and function were altered by developmental exposure to SHS, however these studies did not determine whether SHS exposure during prenatal (*in utero*) or neonatal development had differential effects on adult atherogenesis.

Hence, to determine whether prenatal versus neonatal SHS exposure had distinct effects upon adult CVD development, hypercholesterolemic apolipoprotein E null (apoE−/−) mice were exposed to SHS *in utero* or neonatally (birth to 3 weeks of age) to low dose of SHS (1 mg/m^3^ TSP) and assessed as adults (12–14 weeks of age) for atherosclerotic lesion development, oxidative stress, mtDNA copy number, and mtDNA deletions. For these studies, it was found that both *in utero* and neonatal SHS exposure resulted in significantly increased adult atherosclerotic lesion development and changes within the mitochondrion that heighten CVD susceptibility.

## Materials and Methods

### Ethics Statement

All studies were approved by the University of Alabama at Birmingham Animal Resource Program IACUC (APN 090707224, Assurance Number A3255-01).

### Mice

Apolipoprotein E null (apoE−/−) mice were purchased from Jackson Laboratories (Bar Harbor, ME) and shipped directly to the University of California-Davis (UC Davis) for use as breeders for the SHS exposures. Apolipoprotein E is an important ligand for lipoprotein receptors in the liver, and thus apoE−/− mice have high plasma levels of cholesterol (VLDL, IDL, and LDL are significantly elevated) and triglycerides compared to wild-type mice [16]. The hypercholesterolemic apoE−/− mouse is a widely used model for atherosclerosis, and has been previously shown to sustain increased atherogenesis when exposed to SHS [13], [17]–[19]. Atherosclerotic lesion development in apoE−/− mice has been well characterized with studies showing that they develop atherosclerotic lesions similarly to humans [20], [21]. Diet and water were provided *ad libitum* before and subsequent to, but not during, SHS exposure. Male offspring from pregnant mice were fed chow (PicoLab Rodent Chow 20) diets until 12–14 weeks of age.

### Exposures

**Figure 1:**
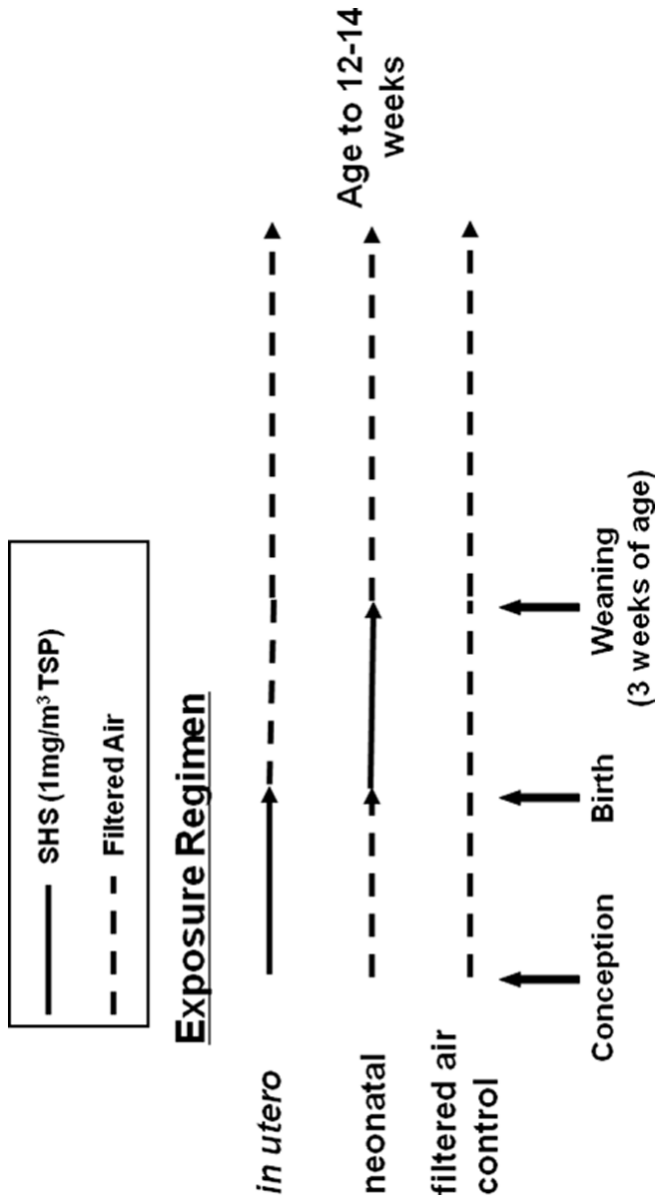
Experimental exposure regimen to second-hand smoke (SHS). apoE−/− mice on a C57BL/6J background were developmentally exposed to either filtered air or 1 mg/m^3^ total suspended particulate (TSP) SHS. The *in utero* group were exposed to 1 mg/m^3^ TSP from gestation days 1–19, followed by filtered air exposure until sacrifice at 12–14 weeks of age. The neonatal group were exposed to 1 mg/m^3^ TSP within 24 hours of birth until weaning at 3 weeks of age, followed by filtered air exposure until sacrifice at 12–14 weeks of age. Control mice were exposed exclusively to filtered air, until sacrifice at 12–14 weeks of age.

All SHS exposures were performed at the Center for Health and the Environment, University of California-Davis in accordance with institutional guidelines. Mice were exposed to *in utero* or neonatal SHS at a concentration of 1 mg/m^3^, 4–5 ppm carbon monoxide, and 200–300 µg/m^3^ nicotine. This dose of SHS falls well within the range for humans observed indoors [22]. Exposures were performed as previously described [13], using a regimen of either: (i) filtered air or 1 mg/m^3^ SHS (6 hrs/day) from gestation days 1–19 (*in utero* exposure), followed by filtered air exposure until sacrifice at 12–14 weeks of age, or (ii) filtered air or 1 mg/m^3^ SHS (6 hrs/day) from birth to 21 days of age (neonatal exposure), followed by filtered air exposure until sacrifice at 12–14 weeks of age (**Figure 1**). There were no significant differences in age between animals used in these studies. Average steady state levels of TSP, carbon monoxide (CO), nicotine, temperature, and percent humidity were (mean±standard deviation): TSP –1.03±0.01 mg/m^3^, CO –3.45±0.15 ppm, nicotine –205.25±52.81 µg/m^3^, temperature –23.19±0.65°C, humidity –18.25±10.75%. All studies were approved by the University of Alabama at Birmingham Animal Resource Program IACUC (APN 090707224, Assurance Number A3255-01).

### Tissue Collection

Mice were anesthetized by interperitoneal injection of Ketastat/Xylazine (60 mg/kg Ketastat/10 mg/kg Xylazine), and exsanguinated. The aortas (from the aortic root to the thoracic aorta, proximal to the iliac artery) were perfused with cold PBS immediately following removal and then flash-frozen (adventitial tissue was removed as much as possible prior to freezing) in liquid nitrogen unless used for oil red-O staining or isolation of mitochondria. Tissues were stored at −80°C until use. Aortas prepared for oil red-o had adventitial tissues removed and were washed with PBS and fixed in formalin.

### Oil Red-O Staining

Whole aortas were stained with oil red-O to assess atherosclerotic plaques as previously described [13], [19]. Briefly, whole aortas were removed, washed in PBS and fixed in buffered formalin overnight. Connective tissues were removed and aortas were cut longitudinally and stained in oil red-O. Aortas were mounted using AquaMount and plaques were quantified utilizing ImageJ, with oil red-O positive staining area expressed as percent of total aortic area. Briefly, Image J was used for image analysis of aortas stained with oil red-O by determining the total aortic area (flattened aortas were traced and area calculated by Image J), followed by quantification of total oil red-O positive area by calibrating the color threshold to calculate areas based on positive lesion staining; hence only lesion areas were quantified. Next, the oil red-O positive staining area was expressed as a percent of the total aortic area (total oil red-O positive area/total aortic area X 100).

### QPCR for Mitochondrial DNA Copy Number and Deletions

Mitochondrial DNA copy number was determined by QPCR of a 163 bp product as previously described [23].To estimate general levels of mtDNA deletions, a modified QPCR protocol was performed as previously described that favors amplification of shorter mtDNA molecules that represent mtDNA deletions [24]. Briefly, genomic DNA was extracted (Qiagen), quantified via fluorescence (PicoGreen, Molecular Probes) and 15 ng of each sample was used for the QPCR. QPCR products were electrophoresed at 90 volts for 2.5 hours onto a 2% agarose gel. The gels were dried and imaged on a Storm 840 imaging system (GE Healthcare). Total mtDNA deletion levels for each sample were estimated by the quantification of the all QPCR products (band intensities for each sample lane) using ImageQuant (GE Healthcare). Estimates of mtDNA deletion frequency (the number of different mtDNA deletions) for each sample was done by counting the number of bands per lane for each individual sample. All analyses for mtDNA copy numbers and deletions were performed in a blinded fashion.

### Aconitase Activity and Immunoblots

Aconitase activity was determined by measuring the transformation of isocitrate to cis-aconitate at 240 nm in 50 mM TrisHCl (pH 7.4) containing MnCl_2_ and 20 mM isocitrate at 25°C. Aconitase is specifically inactivated by superoxide (O_2_ **^.^** ^−^) and peroxynitrite (ONOO^−^) and hence, decreased activity correlates with increased oxidative stress associated with superoxide (O_2_^. −^) and peroxynitrate (ONOO^−^) [25]. Because ONOO^−^ formation is related to O_2_^.−^ production, decreased aconitase activity can be associated indirectly with increased O_2_^.−^ generation.

To evaluate the specific activity of aconitase, western blots were performed using the same homogenates used for the enzyme activity. Decreases in enzymatic activity without significant changes in aconitase protein level were interpreted as consistent with decreased enzymatic specific activity. Antibody specific to aconitase was generated based upon the NCBI reported murine mitochondrial aconitase (Acon2) sequences. After analysis of the protein sequence, three peptides were chosen for conjugation and antigen preparation. These sequences are specific to mitochondrial aconitase and are not reported to be present in cytosolic aconitase:

1. N′-HLDDPANQEIERGKTYLRLRPDR-C′
2. N′-DHLIEAQVGGEKDLRRAKD-C′
3. N′-HRMKKYLSKTGRTDIANLAEEFKDH-C′

Upon synthesis, peptides were each conjugated to KLH via their N′ and their C′ termini, dialyzed, lyophilized and made ready to immunize two rabbits per peptide. Serum was collected at pre-immune, 6 weeks, 8 weeks and 9 weeks. The sera were pooled, screened by ELISA and Western blot, and later affinity purified against the peptides.

To perform immunoblots on homogenized tissue, 50 µg of protein was loaded onto 4–12% Bis Tris PAGE gels (Bio-Rad; Hercules, CA), subjected to electrophoresis, transferred to PVDF membrane, blocked in milk, and immunobloted with the anti-aconitase antibody for two hours. Following incubation with the primary antibody, blots were immunoblotted with secondary HRP-goat anit-rabbit IgG antibody. Blots are visualized using ECL Plus Western Blotting Detection Reagent (Amersham).

### SOD2 Activity and Immunoblotting

Mitochondrial SOD (SOD2) activity and protein levels were determined as previously described [19].

### Statistics

For all endpoints, a minimum of five animals per group were utilized. Results are expressed as mean ± SEM. A Shapiro-Wilk normality test was performed prior to analysis of variance (ANOVA) which tested the null hypothesis that all samples were drawn from a single population. All data within this study passed the normality test. If significant differences (*P*<0.05) existed, then a Student-Newman Keuls test was used for group comparisons. All statistical analyses were carried out using SigmaStat statistical software.

## Results

**Figure 2:**
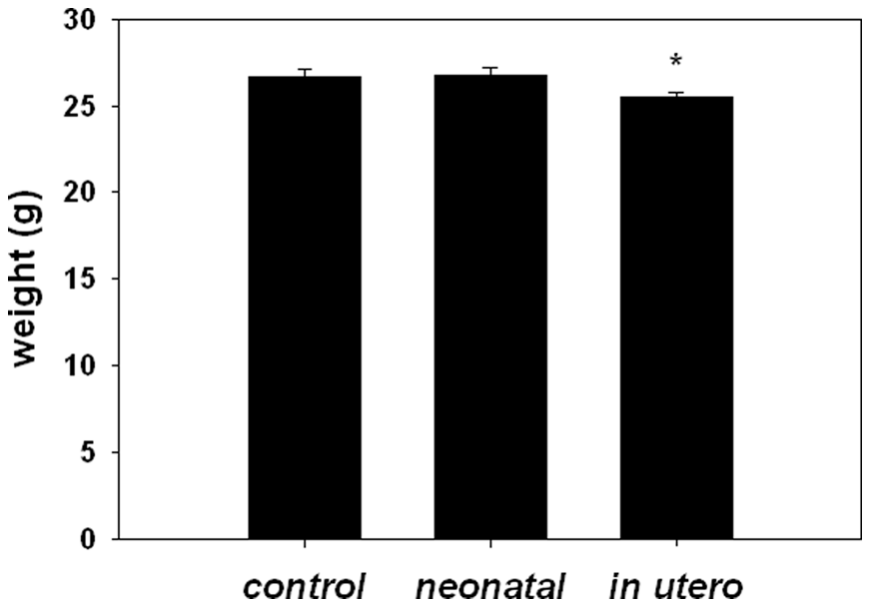
Adult weights from apoE−/− exposed to developmental SHS. Bar graph depicting average weight of mice at 12–14 weeks of age, prior to sacrifice. An asterisk (*) indicates a significant difference (p<0.05) exists compared to control (n = 39, 30, and 19 for control, *in utero*, and neonatal groups, respectively).

No differences were observed between SHS exposed and filtered air control mothers in pregnancy weight or litter sizes, indicating that fertility and viability were not affected by the utilized SHS regimen, as previously noted [13]. Similarly, no differences in total plasma cholesterol of were observed in adult offspring between groups (485+41 mg/dL, 472+34 mg/dL, and 441+41 mg/dL for control, neonatal and *in utero* exposure groups, respectively). By contrast, *in utero* SHS exposure was associated with decreased average body weight in adult (12–14 weeks old) mice compared to both control and neonatal SHS exposed mice (**Figure 2**).

**Figure 3:**
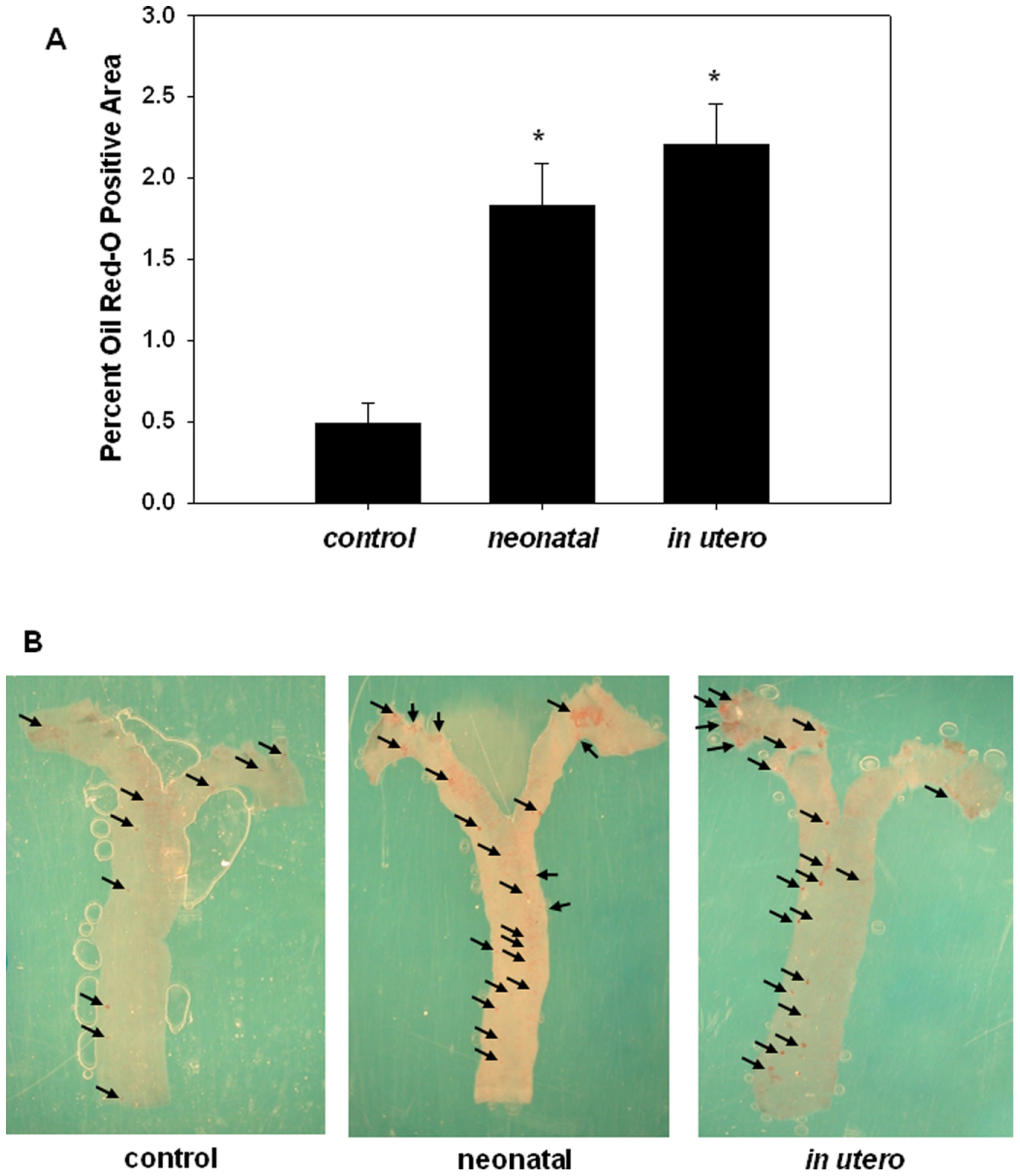
Oil red-O staining of aortas from developmental SHS exposed mice. Atherosclerotic lesion levels were assessed by quantifying the percent of oil red-O staining of whole aortas from mice exposed either to *in utero* or neonatal SHS (1 mg/m^3^ TSP). A) Bar graph demonstrating the percent levels of oil red-O positive staining aortic area relative to total aortic area from control, neonatal, and *in ute*ro exposed apoE −/− mice. An asterisk (*) indicates a significant difference exists compared to filtered air control (p≤0.001; n = 5, 6, and 8 for control, *in utero*, and neonatal groups, respectively). B) *En face* images of oil red-O stained aortas; arrows indicate presence of positively stained area.

Developmental exposure to SHS significantly increased oil red-O staining of whole aortas from 12–14 week old adult apoE−/− mice fed a chow (4.5% fat) diet (**Figure 3**). Percent oil red-O staining area (indicative of atherosclerotic lesion formation) significantly increased with *in utero* or neonatal SHS exposure compared to filtered air controls (**Figure 3**). Differences were not observed in the level of oil red-O staining between *in utero* and neonatal SHS exposed mice, suggesting that in terms of atherogenesis, both developmental exposure periods yielded similar effects on adult atherosclerotic lesion development relative to filtered air controls.

**Figure 4:**
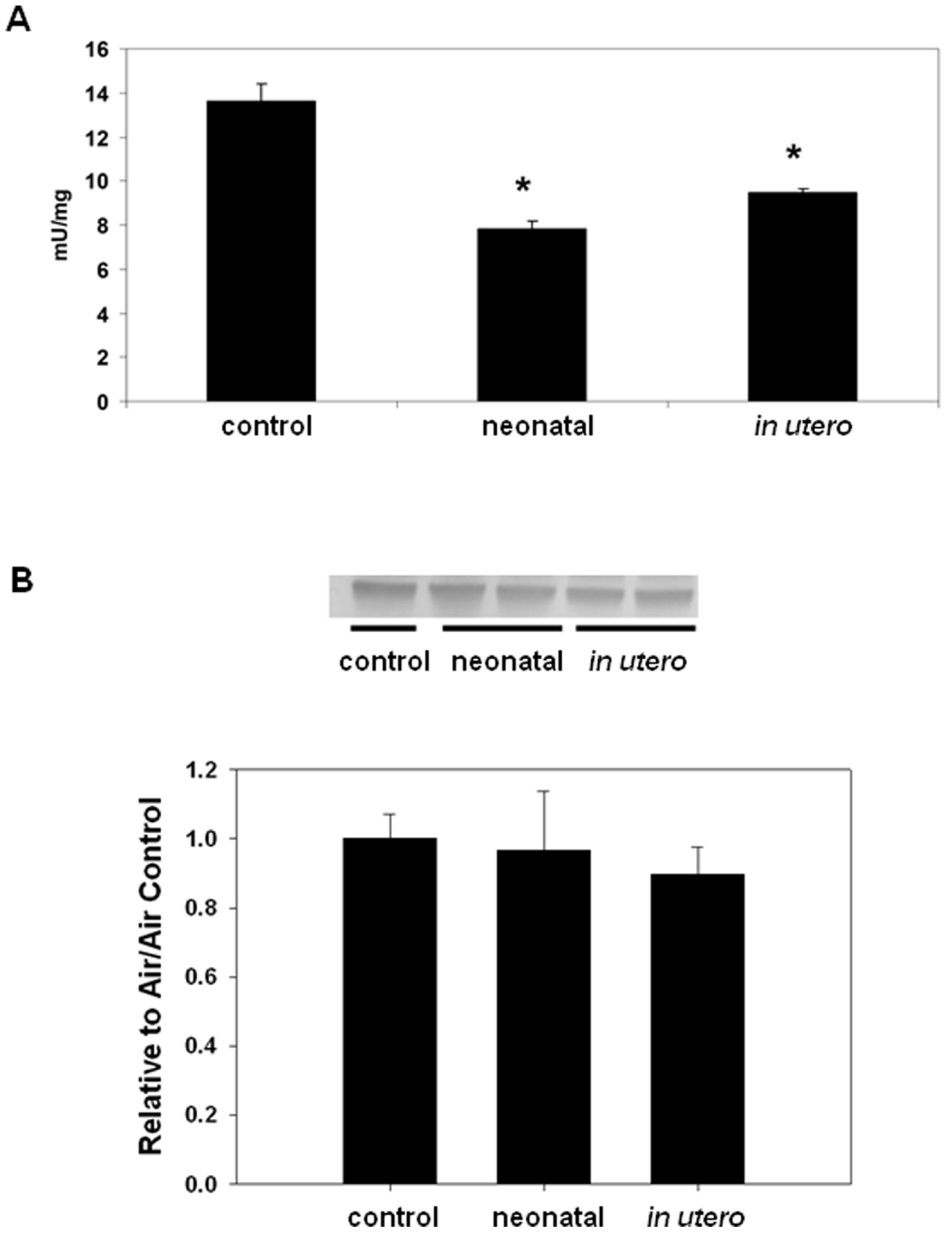
Aconitase activity from aortas harvested from mice exposed to SHS during development. Aconitase is specifically inactivated by superoxide (O_2_^.−^) and peroxynitrate (ONOO^−^) and therefore provides an indirect measure of O_2_^−.^ associated oxidant stress. A) Aconitase activity was measured in aorta homogenates from mice exposed to filtered air, neonatal SHS, or *in utero* SHS by following the production of cis-aconitate from isocitrate at 240 nm. An asterisk (*) indicates a significant difference exists compared to filtered air control (p≤0.001; n = 8/group). B) Immunoblot showing aconitase protein levels; the bar graph below depicts the quantification of relative aconitase protein levels to control (n = 8/group).

Because oxidative stress is known to play an important role in atherosclerosis initiation and progression [26]–[29], [29,30], oxidant load assessment was performed by quantifying aconitase activity in aortic homogenates. Aconitase is a redox sensitive, citric acid cycle enzyme that is specifically inactivated by superoxide (O_2_^.−^) and peroxynitrite (ONOO^−^) [25]. **Figure 4** shows that aortic aconitase activity was significantly decreased in mice exposed to either *in utero* or neonatal SHS compared to filtered air controls; furthermore, aconitase protein levels were not significantly different between exposure groups (**Figure 4**) suggesting that the changes in activity were not due to diminished protein levels between exposure groups. These results are consistent with changes in aconitase enzyme specific activity mediated by increased oxidative stress (O_2_^.−^ and ONOO^−^).

**Figure 5:**
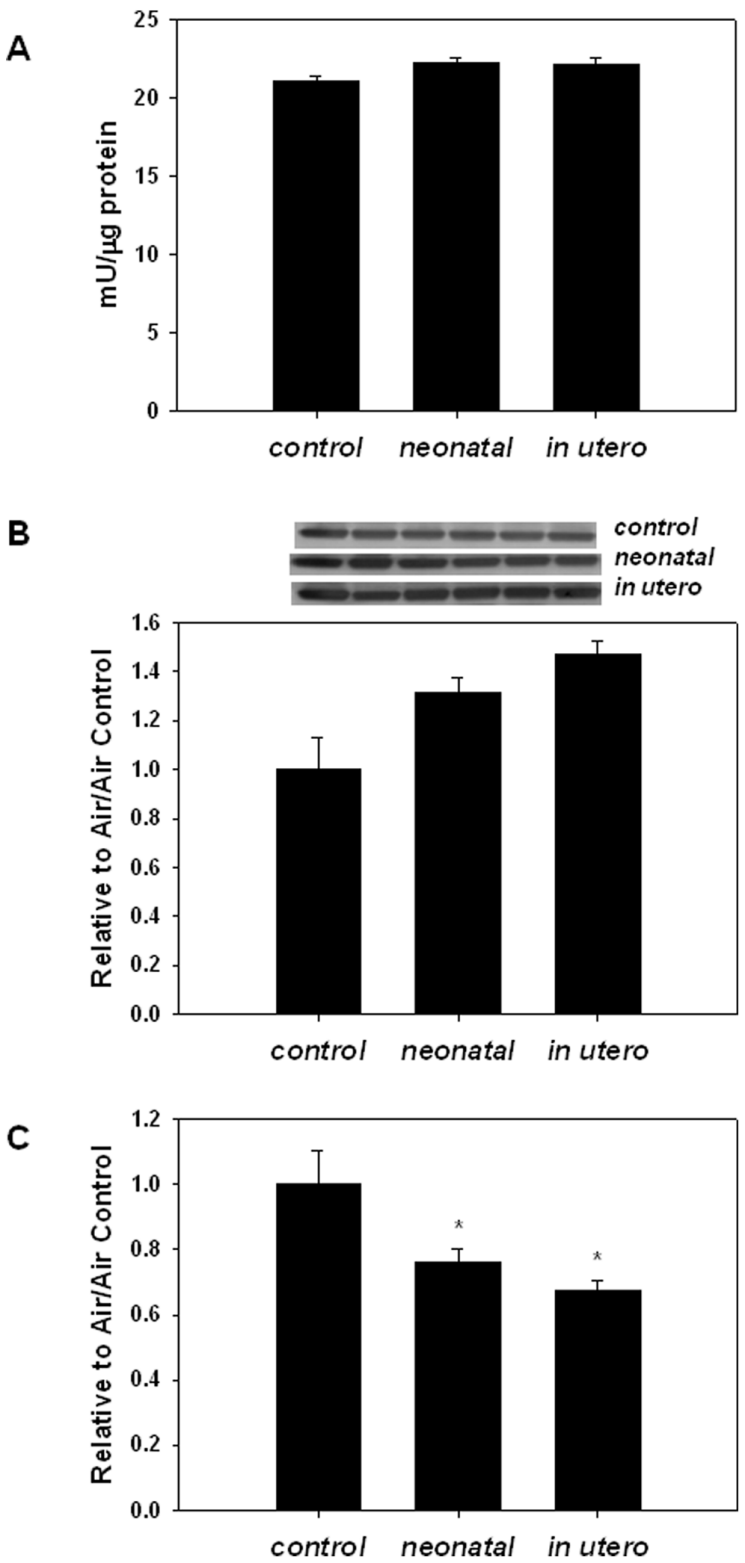
Mitochondrial superoxide dismutase (SOD2) activity, protein levels, and specific activity in aortas harvested from apoE−/− mice exposed to SHS *in utero* or as neonates. Aortic homogenates were utilized to quantify SOD2 enzymatic activity and protein levels from 12–14 week old apoE−/− mice exposed to filtered air (control) or SHS either neonatally or *in utero*. Bar graphs show: A) SOD2 activity, B) SOD2 protein levels; representative immunoblots are shown (all blots were run and immunoblotted together). The first lane for each row is the same sample (vascular homogenate from a SOD2 overexpressor transgenic mouse) and all blots were normalized to that sample. The bar graph depicts relative SOD2 protein levels to control group, and C) SOD2 specific activity in control, neonatal, and *in utero* SHS exposed mice. An asterisk indicates a significant difference (p<0.01; n = 8, 5, and 7 for control, *in utero*, and neonatal groups, respectively) exists from control.

These data were consistent with the notion that developmental SHS exposure increases O_2_^.−^ related oxidative stress in the vasculature. Quantification of mitochondrial superoxide dismutase activity (SOD2 or MnSOD, which catalyzes O_2_^.−^ dismutation) from aortic homogenates showed that while no differences existed in total SOD2 activity, differences were observed in SOD2 protein levels in the adult mice exposed to developmental SHS, also resulting in decreased SOD2 specific activity in adult animals exposed to SHS *in utero* or as neonates (**Figure 5**).

**Figure 6:**
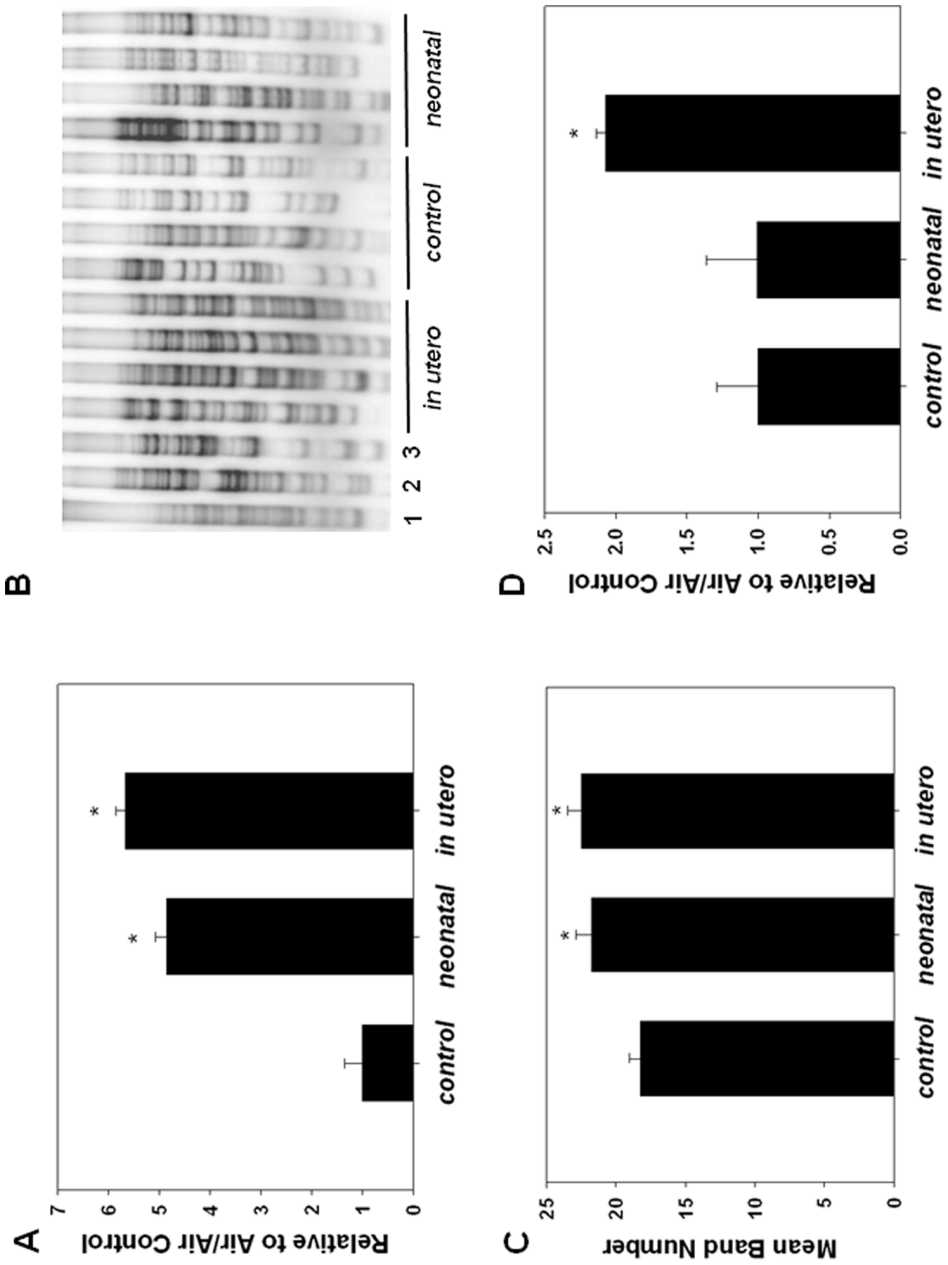
Mitochondrial DNA (mtDNA) copy number and deletion levels associated with developmental SHS exposure. Genomic DNA was extracted from whole aortas and mtDNA copy number and mtDNA deletion levels and frequency were estimated by QPCR. A) MtDNA copy number in control, *in utero* or neonatal exposure apoE−/− mice. Values are expressed relative to control. An asterisk indicates a significant difference (p<0.01; n = 10, 8, and 7 for control, *in utero*, and neonatal groups, respectively) exists from control. B) QPCR results showing mtDNA deletions in the control, neonatal, and *in utero* groups (indicated); lanes 1–3 are from control, *in utero*, and neonatal samples, respectively. Lanes 4–7 are from *in utero* exposed, lanes 8–11 are from control, and lanes 12–15 are from neonatal exposed samples. C) Mean number of different mtDNA deletions per group (represented by a different band per mtDNA deletion), as assessed by QPCR. An asterisk indicates a significant difference (p<0.05; n = 5/per group) exists from control. D) Mean amount of mtDNA deletion levels in aortic tissues from control, neonatal, and *in utero* exposed apoE−/− mice quantified by utilizing QPCR. An asterisk indicates a significant difference (p<0.01; n = 5/per group) exists from control and neonatal.

MtDNA copy number was increased in aortas from mice exposed to *in utero* or neonatal SHS relative to filtered air controls (**Figure 6**), consistent with reports that oxidative stress and exposure to cigarette smoke can up-regulate mtDNA copy number [15], [31]–[34]. To further investigate whether developmental SHS exposure was associated with mutational changes in the mtDNA, overall levels of mtDNA deletions were quantified in each exposure group using PCR analysis as previously described [24]. Interestingly, while the frequency of different mtDNA deletions was significantly increased in adult mice exposed to *in utero* or neonatal SHS compared to controls (**Figure 6C**), the accumulated amount of mtDNA deletions was significantly higher in only the *in utero* exposed SHS animals (**Figure 6D**).

## Discussion

Atherosclerosis is a chronic, pro-inflammatory disease resulting from the accumulation of lipids, macrophages, T-cells, and vascular smooth muscle cells in the sub-intimal space. Cigarette smoke exposure is one of the strongest predictors of CVD development and it has been estimated that exposure to second-hand smoke (SHS) increases the risk of CVD by 30% [1]. However, while many studies utilize SHS exposures that are not physiologically relevant, the study presented herein utilizes a dose of 1 mg/m^3^ TSP, reminiscent of the levels noted in a smoky bar [4], [35]. Exposure of apoE−/− male mice, *in utero* or neonatally, to this dose significantly increased atherosclerosis in adulthood relative to controls to a similar extent for both exposure regimens. This is consistent with several adult animal exposure studies that have shown increases in atherosclerosis burden at higher doses of SHS exposure, such as apoE−/− mice to 30 mg/m^3^ TSP on a chow diet [19], transgenic apoB^100^ adult mice fed Paigen’s atherogenic diet (15% fat, 1.25% cholesterol, 0.5% sodium cholate) exposed to 25 mg/m^3^ TSP [35], and female apoE−/− mice fed a Western Diet (21% fat, 0.15% cholesterol) exposed to 25 mg/m^3^ TSP [17]. While these studies show increased atherosclerosis with adult SHS exposure, they did not address the potential effects of developmental SHS exposure on adult disease development, and most used high fat and high cholesterol diets which make it difficult to discern the effects of diet versus SHS exposure.

The results of this study suggest that while perinatal exposure to SHS indeed significantly influences the development of atherosclerosis in the adult, this sensitivity is not significantly affected by the timing of developmental SHS exposure in apoE^−/−^ mice as both *in utero* and neonatal exposure showed comparative increases in oil red-O staining. Similar effects were observed in the vasculature regarding measures of oxidative stress such as aconitase activity, SOD2 specific activity, mtDNA copy number, and the number of distinct mtDNA deletions. Interestingly, the accumulation of mtDNA deletions was different depending on the timing of the SHS exposure; while *in utero* SHS exposure increased the overall amount of mtDNA deletions, neonatal exposure to SHS was not associated with such an increase in the adult. This difference may be temporal, in that *in utero* SHS exposure contributed to deletion formation at an earlier developmental period compared to neonatal exposure. During embryogenesis rapid growth and cell division occur, and early introduction of a mtDNA deletion will result in a greater accumulation of these deletions over time in the adult tissues. The observation that the number (frequency) of mtDNA deletions is similar between *in utero* and neonatal exposures, yet the levels of mtDNA deletions are higher in the *in utero* animals, is consistent with the increased accumulation of deletions over time [36], [37]. Consequently, we anticipate that mtDNA deletion levels would increase over time in the neonatal SHS exposed apoE^−/−^ animals compared to unexposed controls and eventually become distinct. This may also suggest that it is the number of different types of mtDNA deletions that influence atherogenesis. In addition, empirically it appears that both the *in utero* and neonatal exposure tissues appear to have a higher number of large mtDNA deletions (represented by the smaller PCR products, **Figure 6B**) compared to the control samples. Larger mtDNA deletions would encompass a greater number of mtDNA encoded genes and therefore potentially have a greater impact on cell function.

In aortic homogenates, aconitase, a redox sensitive enzyme, was shown to have decreased activity following an *in utero* or neonatal exposure to SHS suggesting increased oxidative stress. Increases in oxidative stress within the mitochondrion are associated with increased mtDNA damage, redox modification and inactivation of proteins, and overall mitochondrial dysfunction [4], [13], [17], [19], [26], [31]–[35], [38]–[46]. The mtDNA is particularly sensitive to oxidative damage and studies have shown increases in mtDNA damage in relation to CVD development [19], [47], [48]. Chronic ischemia has been shown to increase mitochondrial DNA deletions in humans [49] and CVD patients have been shown to have higher levels of mtDNA damage in heart tissue compared to control patients [50]. Our group has previously shown that mtDNA damage is significantly increased with adult or *in utero* exposure SHS [13], [15], [19] and adult atherosclerosis, and also that increased mitochondrial oxidant stress due to decreased SOD2 activity increases atherogenesis, cardiovascular mtDNA damage, and greater oxidant output under pro-inflammatory conditions [23]. Pre-existant mitochondrial oxidative stress also increased the levels of mitochondrial damage and atherogenesis associated with SHS exposure [23]. Similar to the current study, *in utero* exposure of apoE−/− males to a substantially higher dose of SHS than used here (10 mg/m^3^ TSP vs. 1 mg/m^3^ TSP) has been shown to increase atherogenesis and mtDNA damage [13], and studies investigating the effects of perinatal exposure to SHS in the non-human primate model *Macaca mulatta* have been shown to increase aortic mtDNA damage [15].

During development, mitochondria serve a major role in providing the energy required for the rapid growth associated with this phase in life and may play key roles in signaling [40], [41], [51]–[53]. Environmental factors experienced during this time that result in mitochondrial dysfunction are likely to have long-lasting effects. As discussed, several studies have investigated the role of mitochondrial damage and dysfunction following SHS exposure in various animal models [15], [19], [33], [42]–[44], [48]. Cytochrome c oxidase activity has been shown to be decreased in lymphocytes of chronic smokers [44] and Gvozdjáková *et al.* have shown impairment of oxidative phosphorylation in SHS in exposed rabbit myocardial mitochondria [42], [54]. Morphologically, it has been noted in cardiomyocytes of rabbits and guinea pigs exposed to carbon monoxide (a component of SHS) that the mitochondria appear swollen with a condensed matrix further indicating impaired mitochondrial function [45], [46]. Collectively, these studies clearly indicate that SHS exposure results in impaired mitochondrial function and increased mitochondrial damage. Consequently, we propose that developmental SHS exposure influences individual predisposition for disease development as an adult, including atherosclerosis. A potential mechanism for initiating these processes is mitochondrial damage mediated by perinatal exposure to the constituents of cigarette smoke, thus initiating mitochondrial damage and dysfunction at an early and critical stage in life. Previous reports showing increased atherogenic susceptibility in apoE−/− mice with decreased SOD2 activity and early mtDNA damage in apoE−/− mice prior to or coincident with fatty streak formation are consistent with this notion [23], [48].

### Conclusions

These studies show that both *in utero* and neonatal exposure to a low dose of SHS increases adult atherosclerosis, and that certain molecular similarities exist between these two forms of developmental exposure. By contrast, differences in the levels of mtDNA deletions exist between *in utero* and neonatal exposure, suggesting that developmental stage of exposure can influence levels of mtDNA deletions. However, in absence of a longitudinal study to investigate appearance and progression of atherosclerotic lesion development, these current results also suggest that exposure at different developmental stages can yield similar pathologies in terms of atherosclerosis, and overall suggests that a potentially large window exists during childhood in which environmental exposures can significantly influence adult CVD development and susceptibility. Further studies directed at determining when lesions appear and how they progress will shed light on whether differences exist in these parameters between *in utero* and neonatal SHS exposure. In addition, whether an interaction exists between developmental exposure to SHS and diet (e.g. high fat western diet) remains to be addressed. However, the current study does clearly suggest that both *in utero* or neonatal exposure to a low dose of SHS increases adult atherosclerosis, oxidative stress, and mitochondrial damage.
